# Women, Environments and Chronic Disease: Shifting the Gaze from Individual Level to Structural Factors

**DOI:** 10.4137/EHI.S989

**Published:** 2009-01-23

**Authors:** Natalie Hemsing, Lorraine Greaves

**Affiliations:** 1 Tobacco Research Coordinator, British Columbia Centre of Excellence for Women’s Health, Vancouver, British Columbia, Canada; 2 Executive Director, British Columbia Centre of Excellence for Women’s Health, Vancouver, British Columbia, Canada

**Keywords:** environment, respiratory disease, cardiovascular disease, sex, gender

## Abstract

**Introduction:**

Chronic heart and respiratory diseases are two of the leading causes of morbidity and mortality affecting women. Patterns of and disparities in chronic diseases between sub-populations of women suggest that there are social as well as individual level factors which enhance or impede the prevention or development of chronic respiratory and cardiovascular diseases. By examining the sex, gender and diversity based dimensions of women’s lung and heart health and how these overlap with environmental factors we extend analysis of preventive health beyond the individual level. We demonstrate how biological, environmental and social factors interact and operate in women’s lives, structuring their opportunities for health and abilities to prevent or manage chronic cardiovascular and respiratory diseases.

**Methods:**

This commentary is based on the findings from two evidence reviews, one conducted on women’s heart health, and another on women’s lung health. Additional literature was also reviewed which assessed the relationship between environmental factors and chronic heart and lung diseases. This paper explores how obesogenic environments, exposure to tobacco smoke, and the experience of living in deprived areas can affect women’s heart and respiratory health. We discuss the barriers which impede women’s ability to engage in physical activity, consume healthy foods, or avoid smoking, tobacco smoke, and other airborne contaminants.

**Results:**

Sex, gender and diversity clearly interact with environmental factors and shape women’s promotion of health and prevention of chronic respiratory and cardiovascular diseases. The environments women live in structure their opportunities for health, and women navigate these environments in unique ways based on gender, socioeconomic status, race/ethnicity and other social factors.

**Discussion:**

Future research, policy and programs relating to the prevention of chronic disease need to move beyond linear individually-oriented models and address these complexities by developing frameworks and interventions which improve environmental conditions for all groups of women. Indeed, in order to improve women’s health, broad social and economic policies and initiatives are required to eliminate negative environmental impacts on women’s opportunities for health.

## Introduction: The Burden of Chronic Respiratory and Cardiovascular Diseases

Environmental risk factors, such as tobacco smoke exposure, obesogenic environments and area deprivation, have been linked with a greater risk and poorer management of chronic heart and lung diseases. Evidence from two literature reviews reveals that exposure to environmental tobacco smoke (ETS) increases the risk of coronary heart disease by 30% (Barnoya and Glantz, 2005), and increases women’s risk of dying from heart disease (Kaur, Cohen et al. 2004). Exposure to ETS also increases risk for developing chronic obstructive pulmonary disease (COPD) (British Columbia Ministry of Health 2004), and increases a non-smokers risk of developing lung cancer by 30%–50% (British Columbia Ministry of Health 2004). As well, environmental factors have been shown to contribute to obesity, therefore increasing risk of cardiovascular disease (CVD) and, to some extent respiratory disease (Booth, Pinkston et al. 2005; Cummins and Macintyre, 2006; Lake and Townshend, 2006). Obesogenic environments encourage the consumption of calorie dense but nutrient deficient foods, while inhibiting physical activity. Lastly, living in an area of high deprivation has also been associated with greater rates of smoking, physical inactivity and obesity (Cubbin, Sundquist et al. 2006). As will be shown, women who encounter these environmental risk factors face a greater risk of chronic heart and/or lung diseases.

However, chronic cardiovascular and respiratory diseases are not evenly distributed across all groups. For example, a number of studies conducted in Sweden, New Zealand and the United States have observed an inverse gradient between measures of socioeconomic status (as measured by income and education) and cardiovascular disease risk (Ostrove and Adler, 1999; Tyroler, 1999; Marmot, Shipley et al. 2001; Kuper, Adami et al. 2006; Metcalf, Scragg et al. 2007). In Canada, for example, older persons, Aboriginal people and women have demonstrated greater social disadvantage and more CVD risk factors (Anand, Yusef et al. 2001; Anand, Razak et al. 2006). As well, evidence from Canada and the United States reveals that Aboriginal, South Asian and African American women are at a high risk for CVD (Mensah, Keenan et al. 2002; Witmer, Hensel et al. 2004; Hayes, Denny et al. 2006; Pilote, Dasgupta et al. 2007). Women often have lower socioeconomic status (SES) and less access to healthcare resources than men, so face a greater risk of both heart and respiratory diseases (King and Arthur, 2003; Steele, Richmond-Reese et al. 2006). Women who are living on a low income are both more likely to smoke and live in areas with greater air and water pollution than middle and higher income women, and therefore face a greater risk of developing lung diseases (Greaves and Richardson, 2007).

These patterns of health inequalities suggest that there are differences between women and men, and between sub-populations of women and men, which shape chronic disease and overall health. However, approaches to risk reduction and prevention of disease that make up health promotion programming often focus on factors that manifest as individual level behaviors, such as tobacco reduction or cessation, increasing physical activity, reducing stress and improving diet. These are often referred to as “lifestyle” factors. The focus on “lifestyle” factors has led to widespread attempts to change individual level behavior, without parallel efforts to change social, economic and environmental conditions. Indeed, this approach, when taken alone, can be seen as victim blaming, by placing responsibility for *all* change on the individual. This critique has been made by numerous researchers such as Norma Daykin (1999) who referred to the wider array of factors as creating “landscapes of risk.” Referring to the U.K. strategy, the Health of the Nation, she writes,

“It is often assumed that [health] improvements can be achieved solely through individual changes in lifestyle, including reducing smoking, improvements in diet and increased physical exercise. However, the strategy has been criticized for overlooking the ‘landscapes of risk’ faced by disadvantaged groups (particularly women) in their attempts to secure health and well-being...The health priorities of these groups may reflect day-to-day preoccupations and the need for survival in often difficult environments rather than more abstract and distant risks” (Daykin, 1999).

Still, there continues to be more research on individual level or “downstream” influences on chronic disease development such as obesity, rather than “upstream influences” such as social, economic and environmental determinants of health (Law, Power et al. 2007).

As a contribution to analyzing these upstream influences, this article addresses how sex, gender, diversity and environmental factors interact and operate in women’s lives, contributing to the structuring of their opportunities for health and abilities to prevent or manage chronic cardiovascular and respiratory diseases. We argue for the need to move beyond an individual level of analysis to account for the way biological, social and environmental determinants overlap. In particular, this paper explores how obesogenic environments, exposure to environmental tobacco smoke, and the experience of living in deprived areas can be deleterious to women’s heart and respiratory health. We will discuss barriers which impede women’s ability to access or engage in physical activity, consume healthy foods, and avoid tobacco, others’ tobacco smoke and other airborne contaminants.

## Methods

Two evidence reviews, one on women’s cardiovascular health, and one on women’s respiratory health were conducted, both focusing on literature published between 2000 and 2007. For the cardiovascular health evidence review, we carried out a thorough literature search of the following databases: Embase, PubMed, Academic Search Premier, Cochrane Reviews, Elsevier, Ovid, and Contemporary Women’s Issues. During the search, we utilized a variety of keywords, including: heart health, heart disease, CVD (all kinds including: coronary, cerebral, vascular), sex, women, gender, ethnicity, obesity, hypertension, diabetes, smoking, age, race, SES, psychosocial and stress. Our literature search returned 350 relevant articles, which were reviewed and analyzed for information on sex, gender, and diversity issues associated with women’s heart health.

The respiratory health evidence review involved a search of the following databases for relevant information: PubMed, Academic Search Premier, Embase and Contemporary Women’s Health. We used the following key words: sex or gender in conjunction with pulmonary, respiratory and lung disease/health. Other specific search terms included: COPD, lung cancer, TB, influenza, smoking, pneumonia, and asthma. In total, we reviewed 137 articles with information related to women’s respiratory health, and other relevant documents including grey literature reports. Grey literature was identified through web-based searches, and through consultation with experts in the field. The literature for both of these reviews was narratively synthesized. Selected findings related to sex, gender and diversity based issues associated with women’s heart and lung health were identified and are included in the following commentary.

Finally, additional literature examining the relationship between chronic diseases and the environment was examined, by carrying out searches on PubMed and Academic Search Premier, using the following keywords: sex, gender, women, ethnicity, socioeconomic status (SES), environment, respiratory disease, cardiovascular disease, smoke exposure and obesogenic environments. Twenty additional articles were identified through this search, and are included in this commentary. This literature supplements the findings from the heart and lung health reviews, and informs the exploration of the relationship between gender, chronic disease and the built environment.

## Results

### Sex-based/biological susceptibility to chronic respiratory and cardiovascular diseases

#### Obesity

Obesity is an important risk factor for CVD, and is also somewhat associated with risk and management of respiratory diseases. According to evidence from the Framingham Heart Study, more cases of heart failure in women (14%) than men (11%) can be attributed to obesity (Haslam, 2005). Greater measures of BMI (Hu, Willett et al. 2004) or waist circumference (Lennep, Westerveld et al. 2002; Behan and Mbizo, 2007) have been associated with increased risk for CVD for women. In developed countries such as the U.K., more women than men are exhibiting risky waist circumference measures (Haslam, 2005). Furthermore, women’s risk changes as they age. While men tend to gain more abdominal weight than women prior to menopause, after menopause women’s fat distribution is increasingly localized in the abdominal area (Ley, Lees et al. 1992; Horiuchi, 1997). This may contribute to women’s increased risk of developing CVD post-menopause.

Some evidence indicates that obesity is also associated with greater risk for respiratory disease. In particular, obesity may cause changes in estrogen levels that exacerbate respiratory diseases, such as asthma and COPD, by increasing bronchial hyper-responsiveness (BHR) (Sood, Dawson et al. 2005).

#### Smoke and airborne contaminant exposure

Smoke exposure, both active and passive, is directly associated with the development of both chronic respiratory and cardiovascular diseases. Women may be more susceptible to the damaging effects of smoke on both their lung and heart health. Evidence from Denmark reveals that given the same amount of tobacco use, women are more likely to develop lung diseases earlier and have more severe expression (Prescott, Bjerg et al. 1997). Studies conducted in the U.S., Japan and Taiwan have also found that women who are exposed to similar levels of environmental tobacco smoke (ETS) have a greater risk for developing lung cancer than men (Hirayama, 1981; Bennett, Alavanja et al. 1999; Lee, Ko et al. 2000). Other potential risks to respiratory health include toxic airborne pollutants such as: moulds, nitrous dioxide, formaldehyde, radon and emission from wood burning stoves or the burning of fossil fuels (Public Health Agency of Canada 2007), which have been shown to have either a stronger or similar impact on women, as compared to men in studies conducted in Italy, Taiwan and the U.S. (Biggeri, Pasetto et al. 2004; Chiu, Cheng et al. 2006; Kennedy, Chambers et al. 2007).

Women’s enhanced susceptibility to the respiratory health effects of cigarettes and airborne contaminants may be due to biological factors such as: greater deposition of toxic substances in the lung, impaired clearance of toxins, and/or exaggerated biological responses to these toxins (Sin, Cohen et al. 2007). Some evidence shows that women may have increased bronchial hyper-responsivity when compared with men, which increases women’s risk of developing respiratory diseases (including asthma and COPD), when confronted with smoke and airborne contaminant exposure (Kanner, Connett et al. 1994). Women also have relatively smaller airways in comparison to men, which may enhance their susceptibility to smoke and pollutant exposures (Caracta, 2003). Further, gastrin releasing peptide receptor (GRPR) can cause growth stimulation in lung cancers and may be activated at an earlier stage in women who are exposed to tobacco smoke, either actively or passively (Caracta, 2003). An increased expression of the receptor in females for all levels of smoking has been reported (Caracta, 2003). Lastly, estrogen mediates and potentially enhances the effects of smoke exposure, radon or cooking fumes (Zang and Wynder, 1996; Tang, Rundle et al. 1998; Siegfried, 2001).

Active and passive smoking also increases women’s risk of cardiovascular disease (CVD). Smoking has been identified as a stronger risk factor for myocardial infarction (MI) in women than in men, has been linked with early menopause, and has an unfavorable effect on plasma lipoproteins (Lennep, Westerveld et al. 2002). More than half of MI’s in middle-aged women are due to smoking (Mather, 2004), and relative risk is approximately 50% higher in female smokers compared with male smokers for both MI and all cause mortality (Prescott, Scharling et al. 2002). Evidence also reveals that exposure to ETS increases women’s risk of dying from heart disease (Kaur, Cohen et al. 2004; Barnoya and Glantz, 2005). Some evidence suggests that the anti-oestrogenic effects of smoking may increase women’s susceptibility to heart disease, but further research is required (Prescott, Scharling et al. 2002).

### Gender, diversity and the environment

#### Gender and diversity related patterns of exposure

Gender roles impact how and when women smoke and are exposed to ETS. For the first time, in many parts of the world, young women’s smoking rates are more often equal to or surpassing boys’, contrary to past trends (Wilson, 2000), and women are more often exposed to ETS (Siegfried, 2001). While more men than women report smoking in their workplace, women are more often exposed to ETS in the home (Ernster, Kaufman et al. 2000). Exposure to ETS is widely perceived to be more of a risk for women, due to the lag in smoking trends between men and women resulting in more non-smoking women living with smoking men (Siegfried, 2001). As well, women working at home may be more often subjected to indoor allergens and other airborne contaminants (Harvard Women’s Health Watch, 2003).

Furthermore, women who are living on a low income may have greater risk of being exposed to ETS (Amos, Sanchez et al. 2008). Women living on a low income may be more likely to be exposed to smoke, despite the presence of smoke free policies, and have less capacity to control and limit exposures. For example, Levy et al. (2006) found that smoke-free laws had a larger impact on reducing smoke exposure in medium-education versus low-education females (Levy, Mumford et al. 2006).

Radon, fumes from cooking fuels and heating stoves and SHS are three lung carcinogens to which women are exposed by virtue of spending more time in the home, and are a particular threat in developing countries (Siegfried, 2001). For example, studies have found that women were more exposed to biomass (animal manure, peat, etc) in both China (Ramirez-Venegas, Sansores et al. 2006; Sin, Greaves et al. 2007), and Turkey (Behera and Jindal, 1991; Kiraz, Kart et al. 2003; Wang, Zhang et al. 2005) resulting in respiratory symptoms and diseases. In developed countries, however, these risks can be perceived to be either minimal, or not necessarily gender related (Ernster, 1996). While environmental factors such as smoke and airborne contaminant exposure have significant health impacts, there are additional sex, gender and diversity factors that enhance the role of environmental factors in the development of chronic diseases for women.

#### Obesogenic environments

Environmental conditions may impede women’s ability to engage in physical activity, acquire healthful foods, and therefore manage their weight. Obesogenic environments are described as the promotion of inexpensive, energy dense but nutrient deficient foods, and labor saving devices and under-investment in mass transit (Giskes, Kamphuis et al. 2007). As Chopra indicates, these environmental changes can be linked to global processes, such as global economics and the marketing strategies of large food industry corporations (Chopra and Darnton-Hill, 2004). For example, increasing densities of fast food restaurants, marketing and the low pricing of fatty foods, and larger portion sizing are all part of this process (Block, Scribner et al. 2004; Cummins and Macintyre, 2006; Lake and Townshend, 2006; Giskes, Kamphuis et al. 2007). In addition, sprawling communities, heavy traffic and lack of safety also affect access and ability to engage in physical activity, thereby increasing rates of obesity (Booth, Pinkston et al. 2005; Joshu, Boehmer et al. 2008).

Jilcott and colleagues’ U.S.-based study (2006), identified a number of environmental factors which were associated with women’s health behaviors and capacity to engage in healthy behaviors (Jilcott, Keyserling et al. 2006). For example, women reported the following barriers: lack of restaurants with healthy food choices (41%), lack of farmers markets or fresh produce (50%), not enough affordable exercise facilities (52%), or women appropriate physical activity programs (42%), heavy traffic (47%), and speeding drivers (53%). Overall, women expressed little awareness of affordable exercise venues or nutrition classes. Similarly, in another U.S. study, high risk women who participated in focus groups cited a number of physical factors which challenged their ability to engage in physical activities, including: weather, limited daylight, lack of sidewalks, traffic and distance (Eyler, Vest et al. 2002). Environmental factors structure women’s capacity to engage in preventive health behaviors and may therefore enhance women’s risk of chronic respiratory and cardiovascular diseases.

#### Economically deprived areas

As stated by Kjellstrom et al. (2007), “social determinants often cause their health effects via webs or pathways of environmental exposures” (Kjellstrom, Friel et al. 2007). Clearly, women living on a low income are more likely to live in environments that don’t support healthy living (Chaturvedi, 2003; Mobley, Root et al. 2006). For example, participants in two Swedish studies who were living in the most deprived neighborhoods were at a greater risk for smoking, obesity, and physical inactivity, even after adjusting for individual socioeconomic status (Sundquist, Malmstrom et al. 1999; Cubbin, Sundquist et al. 2006). Similarly, Winkleby et al. (2007) suggest that neighborhoods may contribute to morbidity and mortality from heart disease due to psychosocial stress, limited access to healthy foods and exercise opportunities, and other health care opportunities (Winkleby, Sundquist et al. 2007). Furthermore, women who are living on a low income may encounter different or greater risks due to the interactions between sex, gender, socioeconomic status and environment.

Environmental factors that produce chronic stress may also lead to unhealthy behaviors or impede women from attending to their health (DeSalvo, Gregg et al. 2005). A deprived neighborhood has been characterized as an area with high poverty and low educational attainment, as well as a high concentration of households headed solely by women (Ming, Browning et al. 2007). Such areas often have physical signs of deprivation, such as pollution, noise, run-down housing, litter and few spaces for recreation (Ming, Browning et al. 2007). Moreover, the effect of area depravation may be even greater for women than for men. For example, in an American study by Borders et al. (2006), females with lower income had greater odds of obesity, yet economic disparities in men were not significantly associated with greater odds of obesity (Borders, Rohrer et al. 2006). In one Swedish study which explored gender differences in area effect, women’s risk of developing coronary heart disease (CHD) was 1.9 times higher than for women living in low-deprivation areas; while for men, it was 1.5 times higher (Winkleby, Sundquist et al. 2007). These differences are partially related to gendered roles and patterns which influence how women and men navigate their environment. Safety concern may be a particularly pertinent barrier to women’s health, especially in economically deprived areas. For example, U.S.-based evidence suggests that while women on a low income may be required to be physically active as a form of transport (Ming, Browning et al. 2007), the stress associated with commuting through deprived areas may negate the potential health benefits of being physically active (Bostock, 2001).

Non-white minorities often live in more socially and economically deprived areas, with fewer health resources or opportunities for healthy living (Graham-Garcia, Raines et al. 2001). For example, the consumption of a more energy dense diet is increasingly occurring at lower income levels in the U.S. Block et al. (2004) found that predominantly black and low income neighborhoods in the U.S. had a greater density of fast food restaurants (Block, Scribner et al. 2004). Other U.S.-based research has found that lower income neighborhoods have fewer supermarkets,^1^ more neighborhood grocery stores, more fast food outlets, less fitness facilities and higher obesity rates than higher income areas (Morland, Wing et al. 2002). There are also numerous structural barriers to health for women living in rural areas, including: poverty, access to health care providers, health infrastructure and social isolation (Taylor, Hughes et al. 2002). A study by Chikani and colleagues (2005) found that women living in rural areas in the U.S. were at greater risk for coronary heart disease (CHD) (exhibiting more obesity, poorer diet, and higher blood pressure) (Chikani, Reding et al. 2005).

Finally, area deprivation increases morbidity and mortality risk, even for women and men with above average income or education who are living in deprived areas (Winkleby, Sundquist et al. 2007). Although the mechanisms by which area level influences health are unclear, evidence suggests that area level has significance beyond even individual level indicators of socioeconomic status (SES). A U.S. study by Ming et al. (2007) found that the effects of neighborhood SES had a stronger effect than household income in influencing physical activity (Ming, Browning et al. 2007). Furthermore, they found that women’s physical activity was more responsive to neighborhood context, when compared to men. Overall, these findings reveal that sex, gender and diversity overlap and interact with the environment in multiple ways, often increasing heart and lung health risks for women, particularly sub-populations of women.

## Discussion

Sex, gender and diversity clearly interact with the environment, and shape women’s health, health promotion and the prevention of chronic respiratory and cardiovascular diseases. Not only does the physical space we live in structure our opportunities for health, but women and various sub-populations of women, as shown, must navigate these environments in unique ways based on gender, socioeconomic status, race/ethnicity and other social factors. As well, evidence demonstrates that women face a greater risk of chronic respiratory and cardiovascular diseases due to biological, or sex-based, factors which may increase the impact of the environment on women’s health. Therefore, future research, policy and programs relating to the prevention of these chronic diseases, as well as to intervention design, needs to move beyond linear individually-oriented models. These complexities could be addressed by developing frameworks and interventions which seek to improve environmental conditions along with responding to women’s unique biological conditions.

Further, changes in social and economic policies and initiatives will also be required, as women’s environments and hence, their opportunities for health, are constructed by the socio-economic conditions in which they live. Wisdom et al. (2005) point out that social and economic circumstances play a key role in determining health status, particularly for women (Wisdom, Berlin et al. 2005). They argue that such factors are mediated by government policies. Therefore, improved preventive approaches will require action on multiple levels, from individual to broad social and economic levels, in order to cover and ameliorate the multiple risks discussed here.

Lee argues that environmental policy and public health policy need to be aligned in order to improve health for all, including low income and disadvantaged women and men (Lee, 2002). Robert also argues for the need for research and programming which examines the relationship between the environment and individual health, particularly within areas of high socioeconomic deprivation (Robert, 1999). Robert suggests that community socioeconomic status shapes the health of individuals by impacting both the SES of individuals, as well as through a pathway independent of the individual (Robert, 1999). Environmental factors (both social and physical) influence the health of individuals, independent of their own socioeconomic position. He contends that the gender, class and race of individuals also influence the relationship between environmental context and individual health (Robert, 1999).

Yet, both prevention initiatives and interventions continue to focus on individual level risk factors, rather than addressing the socio-structural factors implicated in women’s health and prevention of chronic disease. For example, evaluations of an intervention with high-risk uninsured women in the U.S. to reduce risk factors associated with heart disease, revealed that substantially more environmental level initiatives and interventions are needed (Yancey, 2004). Further research and subsequent intervention development is required to examine the relationships between sex, gender, diversity and obesogenic environments, in order to develop multi-level strategies which can have lasting health benefits for all sub-populations of women.

Specifically, there is a clear need for more multi-level analyses and interventions addressing community barriers, including strategies such as: efforts to identify and establish safe and convenient walking venues, affordable gyms, culturally appropriate physical activity programs, and identifying and supporting restaurants with healthy options. In particular, Yancey recommends strategies and policies such as: improving areas for safe walking, building coalitions to bring farmers markets to less affluent neighborhoods, educating legislators about public polices that can encourage healthy lifestyle behaviors, and promoting nutritional labeling (Yancey, 2004). King (2003) also argues for the need for large, multilevel environmental and policy level public health approaches to increase women’s physical activity (King, 2000).

Sex, gender and diversity sensitive tobacco policies and smoking cessation programs are required to protect women from the deleterious effects of exposure to environmental tobacco smoke. While legislation intended to decrease such exposure is on the rise, there has been little consideration of how these policies affect women (Ashley and Ferrence, 1998) or even more broadly, men and women, differentially. Further research, policy and program development is required which examines the relationship between gender, diversity, the environment and exposure to tobacco smoke.

The complex relationship between these types of factors and women’s biology, in the context of ethnocultural influences, individual psychosocial issues, health behaviors and empowerment needs requires further study with more advanced theory and methods, in order to identify the mix of factors that ultimately positively affect women’s health behaviors. Moving beyond an individual analysis to consider the issues of sex, gender, and diversity in the context of environmental barriers to women’s heart and lung health will help to improve the health of *all* women, along with the communities and environments in which they live.

## Footnotes

1 This is important considering that supermarkets carry more “heart healthy” food options than neighborhood grocery stores Law, C., C. Power et al. (2007). *Obesity and health inequalities*, 8:19–22.
